# Supplementary figures and images for: Gut Microbiome dysbiosis and immune activation correlate with somatic and neuropsychiatric symptoms in COVID-19 patients
Source: J Transl Med. 2025 Mar 14;23:327. doi: 10.1186/s12967-025-06348-y (PMC11907868; doi:10.1186/s12967-025-06348-y)

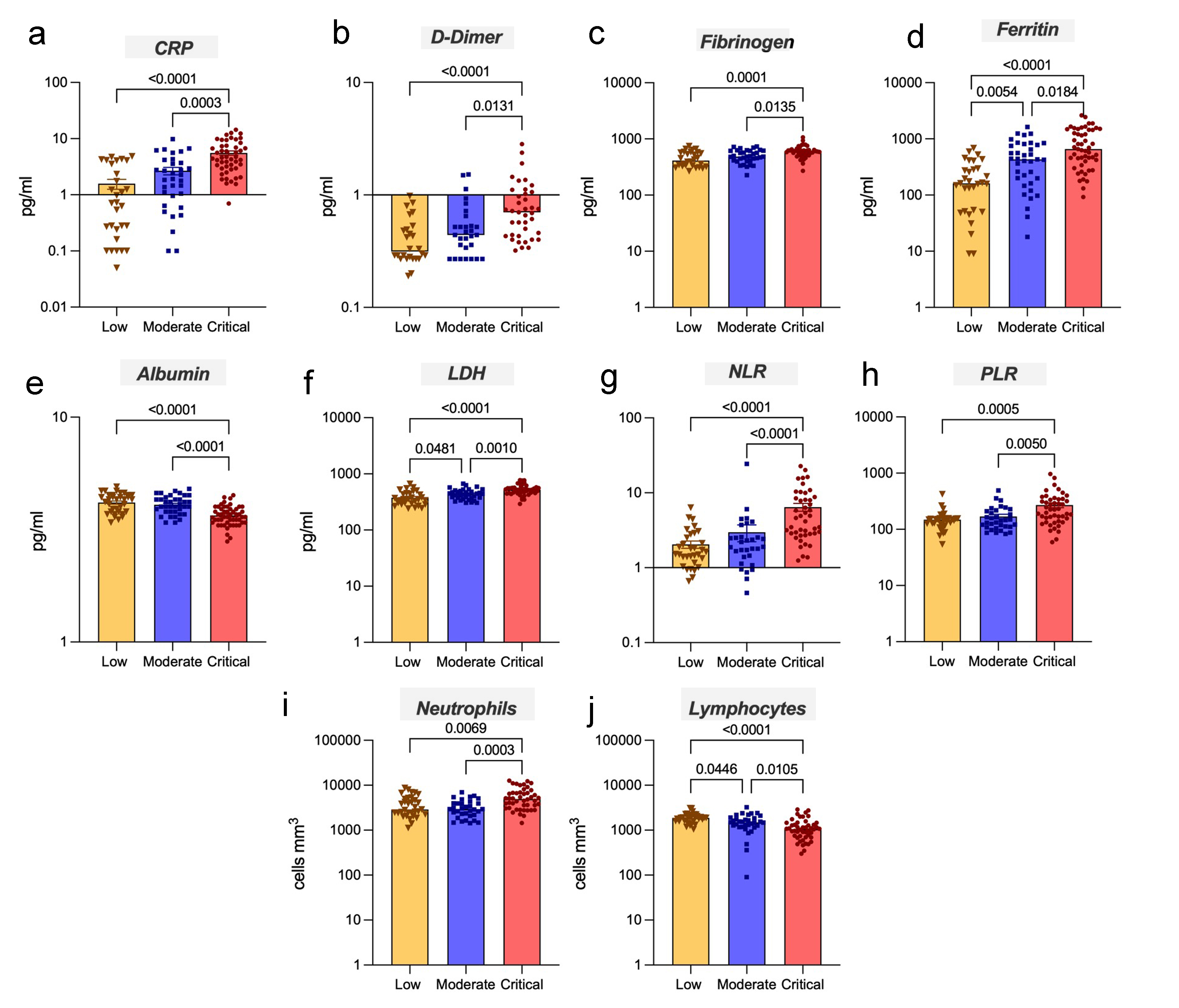

Supplement: Supplementary file 1 — Supplementary Material 1: Fig. S1. Cytokine and chemokine levels across COVID-19 severity groups. Heatmap depicting the relative concentrations of cytokines and chemokines in patients with low, moderate, and critical COVID-19 severity. Significant differences between severity groups are marked (*p < 0.05, **p < 0.01, ***p < 0.001). Elevated levels of IL-1ra, IL-6, IL-10, IL-12, IL-15, and MIP-1a are observed in moderate and critical cases, highlighting their role in systemic inflammation and disease progression [file 12967_2025_6348_MOESM1_ESM.jpg]

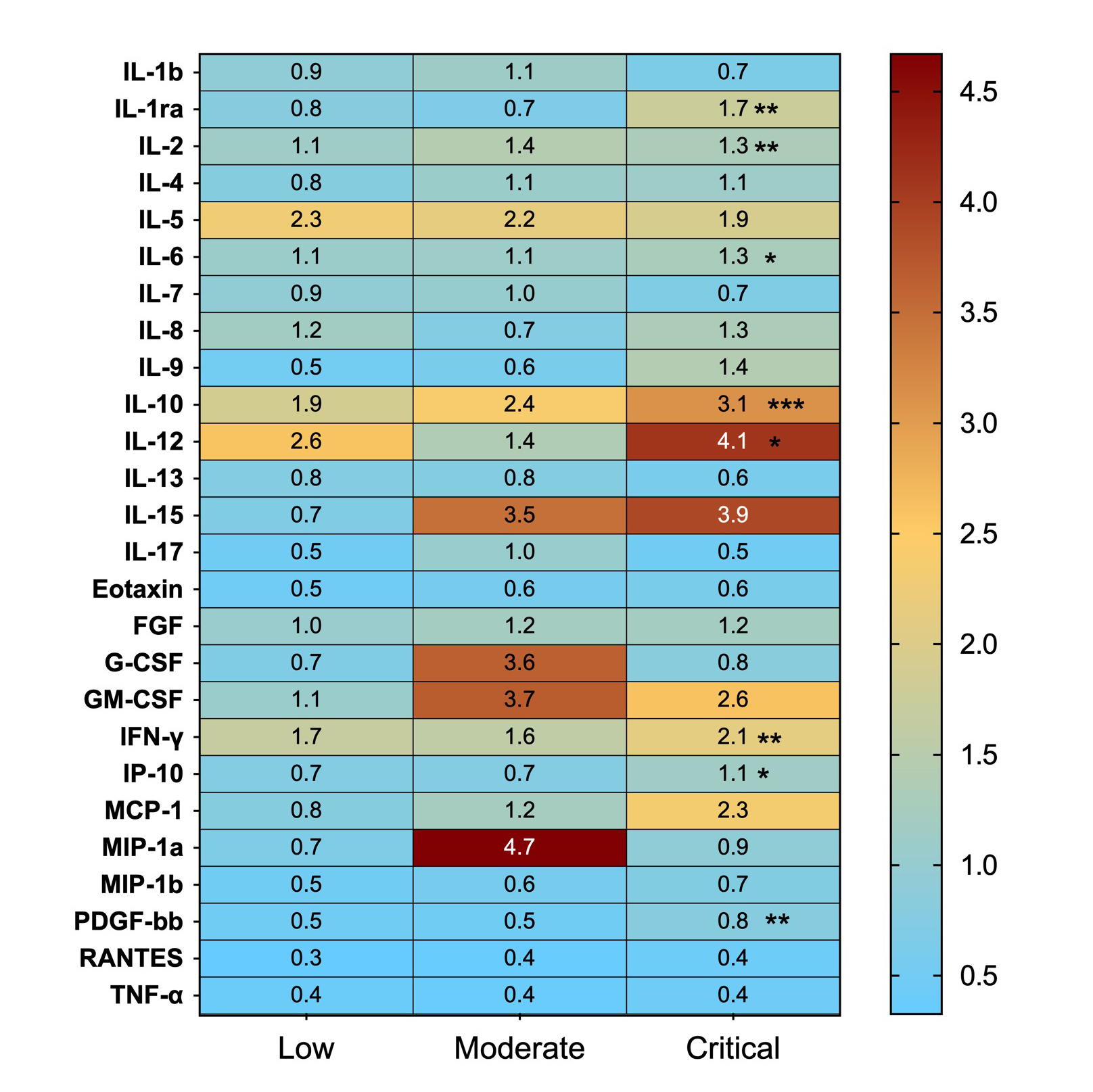

Supplement: Supplementary file 2 — Supplementary Material 2: Fig. S2. Alterations in inflammatory and hematological markers in hospitalized COVID-19 patients across different severity groups. The levels of (a) CRP, (b) D-dimer, (c) Fibrinogen, as well as (d) Neutrophil counts, (e) Neutrophil-to-lymphocyte ratio (NLR), (f) Platelet-to-lymphocyte ratio (PLR) were elevated in patients from the critical group compared to those in the low and moderate groups. Besides the elevated levels in the critical group compared to the low and moderate groups, the levels in the moderate group were also higher compared to the low group for the concentrations of (g) Ferritin, and (h) Lactate dehydrogenase (LDH). Conversely, (i) Albumin levels decrease with increasing severity, with significant reductions in the critical group compared to low and moderate groups. (j) Lymphocyte counts decrease with increasing severity, showing lower counts in critical patients compared to patients in low and moderate groups. These values were lower in moderate compared to low group. ANOVA was followed by Šidak multiple comparisons test or Kruskal-Wallis test was followed by pairwise comparisons between the severity groups. The lines and p-values on the Fig.s indicate significant differences between these groups [file 12967_2025_6348_MOESM2_ESM.jpg]
