# Supplementary material for: Gut Microbiome dysbiosis and immune activation correlate with somatic and neuropsychiatric symptoms in COVID-19 patients
Source: J Transl Med. 2025 Mar 14;23:327. doi: 10.1186/s12967-025-06348-y (PMC11907868; doi:10.1186/s12967-025-06348-y)
Supplement: Supplementary file 3 — Supplementary Material 3 [file 12967_2025_6348_MOESM3_ESM.docx]

**Table S1**. COVID-19 Index Severity.

| **Variables** | | **Scores** |
| --- | --- | --- |
| **Demographic data** | Age (years) | ≤60 = 0  61-64 = 1  ≥65 = 2 |
|  | Gender | Female = 0 / Male = 1 |
| **Chronic diseases** | Heart failure | No = 0 / Yes = 1 |
|  | COPD | No = 0 / Yes = 1 |
|  | Diabetes mellitus | No = 0 / Yes = 1 |
| **Vital signs** | Heart rate (beats per minute) | ≤40 = 3  41 – 50 = 1  51 – 90 = 0  91 – 110 = 1  111 – 130 = 2  ≥131 = 3 |
|  | Respiratory rate (breaths per minute) | ≤8 = 3  9 – 11 = 1  12 – 20 = 0  21 – 24 = 2  ≥25 = 3 |
|  | Systolic blood pressure (mmHg) | ≤90 = 3  90 – 219 = 0  ≥220 = 3 |
|  | Temperature (°C) | ≤35 = 3  35.1 – 35.5 = 1  35.6 – 37.9 = 0  38 – 39 = 1  ≥39.1 = 2 |
|  | Oxygen saturation (%) | ≤91 = 3  92 – 93 = 2  94 – 95 = 1  ≥96 = 0 |
|  | Oxygen saturation in patients with COPD (%) | ≤83 = 3  84 – 85 = 2  86 – 87 = 1  ≥88 = 0 |
| **Laboratory tests** | D-dimer (ng/ml) | ≤1000 = 0  >1000 = 1 |
|  | Lymphocytes (per mm^3^) | ≥1000 = 0  <1000 = 1 |
|  | Platelets (per mm^3^) | ≥10000 = 0  <10000 = 1 |
| **Imaging test** | Chest X-ray | Normal = 0  Bilateral infiltration = 1 |
| **Clinical conditions** | Dyspnea | No = 0 / Yes = 2 |
|  | Supplement oxygen | No = 0 / Yes = 3 |

Predictive variables of worse outcome at hospital admission. Scores of 0-2 indicates low severity, 3-4 moderate, 5-7 high, and 8 or above indicates critical COVID-19 (Adapted from Huespe et al., 2020). COPD: Chronic obstructive pulmonary disease.

**Table S2.** Hematological, coagulation, inflammatory, and biochemical parameters of the patients classified according to the COVID-19 Severity Index (n=124).
