# Supplementary material for: Gut Microbiome dysbiosis and immune activation correlate with somatic and neuropsychiatric symptoms in COVID-19 patients
Source: J Transl Med. 2025 Mar 14;23:327. doi: 10.1186/s12967-025-06348-y (PMC11907868; doi:10.1186/s12967-025-06348-y)
Supplement: Supplementary file 4 — Supplementary Material 4 [file 12967_2025_6348_MOESM4_ESM.docx]

| **Variables** | **All patients (n=124)** |  | **COVID-19 Severity** |  |  |
| --- | --- | --- | --- | --- | --- |
|  |  | **Low**  **(n=34)** | **Moderate**  **(n=37)** | **Critical**  **(n=53)** | **p Value** |
| Hematological parameters |  |  |  |  |  |
| *Leukocytes (cells/μL)* | 5660 (4690 – 8120) | 5640 (4750 – 8205) | 5045 (4505 – 7078) | 6430 (4765 – 9410) | 0.085 |
| *Neutrophils (cells/μL)* | 3825 (2513 – 5438) | 2880 (2330 – 5040) | 2930 (2135 – 3955) | 4955 (3115 – 6808) | 0.0001 |
| *Neutrophils / Lymphocytes ratio (NLR)* | 2.5 (1.5 – 4.6) | 1.6 (1.2 – 2.7) | 1.9 (1.4 – 2.6) | 4.6 (2.8 – 8.6) | <0.0001 |
| *Lymphocytes (cells/μL)* | 1460 (1040 – 1900) | 1880 (1590 – 2135) | 1495 (1178 – 1858) | 1055 (710 – 1423) | <0.001 |
| *Platelets (nº/μL)* | 242927 ± 79063 | 268647 ± 82137 | 230892 ± 66402 | 234673 ± 82824 | 0.080 |
| *Platelets / Lymphocytes ratio (PLR)* | 157 (121 – 245) | 147 (116 – 157) | 145 (110 – 220) | 224 (140 – 342) | 0.0002 |
| Coagulation parameters |  |  |  |  |  |
| *Prothrombin time (s)* | 90.1 ± 9.9 | 89.3 ± 9.3 | 91.1 ± 10.2 | 89.9 ± 10.4 | 0.762 |
| *Activated partial thromboplastin time (s)* | 41.7 ± 6.1 | 41.8 ± 5.9 | 41.7 ± 5.9 | 41.7 ± 6.3 | 0.995 |
| *D-Dimer (μg/mL)* | 0.48 (0.34 – 0.76) | 0.32 (0.27 – 0.50) | 0.44 (0.31 – 0.59) | 0.70 (0.43 – 1.06) | <0.0001 |
| *Fibrinogen (mg/L)* | 548 (418 – 625) | 412 (330 – 576) | 484 (406 – 616) | 599 (516 – 643) | 0.0001 |
| Inflammatory parameters |  |  |  |  |  |
| *C-reactive protein (mg/L)* | 2.93 (1.05 – 4.85) | 0.72 (0.22 – 3.67) | 1.92 (0.80 – 3.95) | 4.49 (2.94 – 7.45) | <0.0001 |
| *Ferritin (ng/mL)* | 433 (176 – 811) | 162 (51 – 296) | 433 (181 – 796) | 659 (399 – 1448) | <0.0001 |
| Biochemical parameters |  |  |  |  |  |
| *Albumin (g/dL)* | 3.93 ± 0.44 | 4.17 ± 0.40 | 4.08 ± 0.38 | 3.65 ± 0.36 | <0.0001 |
| *Creatine kinase (UI/L)* | 94 (56 – 152) | 76 (54 – 135) | 98 (60 – 169) | 107 (48 – 156) | 0.372 |
| *Lactate dehydrogenase (UI/L)* | 456.4 ± 119.7 | 376.4 ± 107.7 | 438.5 ± 92.5 | 523.5 ± 109.6 | <0.0001 |

Note: The data were presented as median (interquartile range) or mean and standard deviation. ANOVA was followed by Šídak multiple comparisons test for parametric parameters or Kruskal-Wallis test was followed by pairwise comparisons between groups.
