# Supplementary material for: Gut Microbiome dysbiosis and immune activation correlate with somatic and neuropsychiatric symptoms in COVID-19 patients
Source: J Transl Med. 2025 Mar 14;23:327. doi: 10.1186/s12967-025-06348-y (PMC11907868; doi:10.1186/s12967-025-06348-y)
Supplement: Supplementary file 5 — Supplementary Material 5 [file 12967_2025_6348_MOESM5_ESM.docx]

**Table S3.** The presence (1) and absence (0) of driver species between COVID-19 severity levels were identified using BakDrive.

| **Bacteria Taxa** | **Low** | **Moderate** | **Critical** |
| --- | --- | --- | --- |
| *Akkermansia muciniphila* | 1 | 0 | 0 |
| *Blautia obeum* | 1 | 1 | 0 |
| *Dorea formicigenerans* | 1 | 1 | 0 |
| *Enterococcus faecium* | 1 | 1 | 0 |
| *Escherichia coli* | 1 | 1 | 1 |
| *Faecalibacterium prausnitzii* | 1 | 1 | 1 |
| *Lactobacillus ruminis* | 1 | 0 | 0 |
| *Paraprevotella clara* | 1 | 0 | 0 |
| *Roseburia inulinivorans* | 1 | 1 | 1 |
| *Ruminococcus bromii* | 1 | 0 | 1 |
| *Streptococcus salivarius* | 1 | 0 | 0 |
| *Anaerostipes hadrus* | 1 | 0 | 0 |
| *Coprococcus catus* | 1 | 0 | 0 |
| *Coprococcus eutactus* | 1 | 0 | 0 |
| *Mitsuokella jalaludinii* | 1 | 0 | 0 |
| *Bacteroides faecis* | 0 | 1 | 0 |
| *Flavonifractor plautii* | 0 | 1 | 0 |
| *Lactobacillus mucosae* | 0 | 1 | 0 |
| *Megamonas funiformis* | 0 | 1 | 0 |
| *Succinivibrio dextrinosolvens* | 0 | 1 | 0 |
| *Bacteroides cellulosilyticus* | 0 | 0 | 1 |
| *Blautia producta* | 0 | 0 | 1 |
| *Blautia wexlerae* | 0 | 0 | 1 |
| *Granulicatella elegans* | 0 | 0 | 1 |
| *Megamonas hypermegale* | 0 | 0 | 1 |
| *Ruminococcus bicirculans* | 0 | 0 | 1 |
| *Ruminococcus champanellensis* | 0 | 0 | 1 |
| *Streptococcus thermophilus* | 0 | 0 | 1 |
| *Streptococcus sanguinis* | 0 | 0 | 1 |
